# Evaluating Aversion to Eye‐Like Stimuli as a Foraging Deterrent in Urban European Herring Gulls

**DOI:** 10.1002/ece3.73202

**Published:** 2026-03-08

**Authors:** Laura A. Kelley, Rosa Hunter Thompson, Beth Rowe, Neeltje J. Boogert

**Affiliations:** ^1^ Centre for Ecology and Conservation University of Exeter Penryn UK

**Keywords:** deterrent, eyespots, habituation, human‐wildlife conflict, kleptoparasitism

## Abstract

Human‐wildlife conflict is on the rise due to urbanisation, and the development of non‐invasive deterrents can help to mitigate negative interactions. European herring gulls *Larus argentatus* are increasingly moving into urban areas, bringing them into conflict with humans. Many animals exhibit aversive behaviour to eyes and directed gaze, and we tested whether gulls foraging in urban areas were deterred by and/or habituated to artificial eye‐like stimuli (known as eyespots) in the short term. We also tested whether aversion to eye‐like stimuli may be due to shape or contrast by testing aversion to high contrast circles and squares. We found that some gulls were slower to approach and less likely to peck a takeaway food box with eye‐like stimuli compared to a box without eyes. When we presented individual gulls with boxes either with or without eye‐like stimuli over three trials, the response to eyes appeared to be individual‐specific. Approximately half of the birds tested consistently avoided boxes with eyes and never approached, indicating a lack of short‐term habituation. The other half approached and pecked at them just as quickly as they did boxes without eyes, suggesting that eyes are unlikely to deter all gulls. There was no difference in approach time or peck likelihood when gulls were presented with circular or square high contrast stimuli, indicating that contrast may be important in eliciting aversion. Overall, our results suggest that high contrast stimuli can deter gulls, although responses appear to be highly individually specific. High contrast and/or eye‐like stimuli may therefore offer a potential tool to help mitigate negative interactions between humans and opportunistic wildlife such as urban herring gulls.

## Graphical abstract

We tested whether eye‐like and high contrast patterns on takeaway food boxes deterred urban herring gulls from approaching and pecking at food boxes. Gulls were slower to approach and less likely to peck at boxes with eyes, and the deterrent effect persisted in the short‐term for some gulls. When used alongside other deterrents, eye‐like stimuli may be an effective tool to negative human–gull interactions.

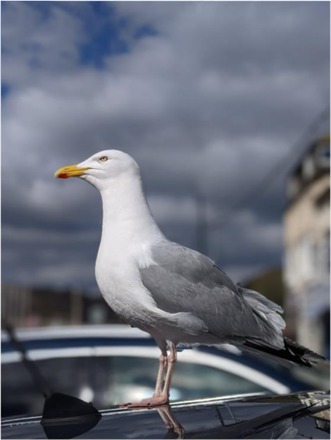

## 1 Introduction

Human‐wildlife conflict arises when interactions between people and wild animals result in negative outcomes for either party (Madden 2004). Conflicts are typically driven by competition over resources such as food and space, often due to a loss of natural habitat and expansion of urban areas. Strategies to mitigate human‐wildlife conflicts may involve deterrents to repel animals from particular areas. To be effective, deterrents need to either create physical barriers, such as fences or crop netting to keep out birds and mammals or employ stimuli that exploit animals' differing behavioural responses. Sensory deterrents induce aversive behaviour, which can be elicited by stimuli that disrupt the senses, such as high frequency sounds (Good et al. 2022; Crawford et al. 2018) or flashing lights (Lesilau et al. 2018). Deterrents can also increase an animal's perception of threat (Schakner and Blumstein 2013) by using conspecific alarm calls (Ribot et al. 2011), predator scent (Valenta et al. 2021) or predator silhouettes (Wang et al. 2010). For instance, auditory deterrents like the playback of the sound of a disturbed bee nest have been used to discourage African elephants *Loxodonta africana* from raiding crops (King et al. 2007). Although many deterrents are developed to prevent agricultural damage caused by crop‐raiding species due to their significant economic impact, they also hold promise as a non‐lethal method for managing wildlife in urban areas. To date, only a handful of studies have investigated the efficacy of sensory deterrents on birds in urban spaces, and tests of visual deterrents have been primarily limited to using bright lights and people physically approaching birds (Calladine et al. 2006; Soldatini et al. 2008; Lecker et al. 2015).

Eye‐like stimuli (also known as eyespots) are generally poorly defined, but typically comprise concentric rings of contrasting colours (typically a dark ‘pupil/iris’ surrounded by lighter ‘sclera’), which occur in bilaterally symmetrical pairs (Stevens 2005). They have been tested in a range of contexts, including to deter ambush predators from attacking cattle (Radford et al. 2020), discouraging birds near airports (Hausberger et al. 2018), to deter birds from foraging (Fukuda et al. 2008; McLennan et al. 1995) and to deter seabirds foraging near gillnets (Rouxel et al. 2021). Aversion to eyes and eye‐like stimuli was first recorded over a century ago (Poulton 1890) and has been documented in humans (Dear et al. 2019) and nonhuman animals alike (Coss 1979; Burger et al. 1991; Hampton 1994; Carter et al. 2008; Davidson et al. 2014). Eye‐like stimuli generally elicit vigilance or avoidance behaviour, which has been attributed to an inherent association with either predators or conspecific competitors (Stevens 2005). Eye‐like stimuli may also be effective due to their high contrast rather than their resemblance to eyes, as many animals exhibit a pre‐existing aversion to high‐contrast conspicuous markings (Mizuno et al. 2024; Stevens et al. 2008; Halpin et al. 2020). Movement may also enhance the efficacy of eye‐like stimuli as they may signal an approaching predator: ‘looming’ or differently sized pairs of eyes on opposite sides of a rotating panel (to create the appearance of looming or receding in quick succession) induced aversive responses in birds (Hausberger et al. 2018; Rouxel et al. 2021). However, efficacy may be species‐specific within a context: ‘looming’ eye‐like stimuli deterred long‐tailed ducks ( *Clangula hyemalis* ) from foraging near gillnets, but not other seabird species (Rouxel et al. 2021, 2023). It is also unclear whether stimuli need to resemble eyes to be effective deterrents or whether the presence of high‐contrast conspicuous markings is sufficient to trigger an aversive response, regardless of their shape (Stevens et al. 2008). Understanding the perceptual mechanisms that make stimuli aversive is critical to their development as effective deterrents.

Here, we investigate whether eye‐like stimuli can act as a deterrent to wild European herring gulls ( *Larus argentatus* , herring gull hereafter) foraging in towns. Although herring gulls are a species of conservation concern in the UK (Stanbury et al. 2021), numbers occurring in urban areas are increasing due to the availability of food and suitable nesting sites, where they can come into conflict with people due to food stealing, nesting on roofs, noisiness and fouling (Calladine et al. 2006; Rock 2005). Herring gulls can use human behavioural cues when foraging; for example, they preferentially approach and/or peck at food or other anthropogenic objects that a person has handled (Goumas, Boogert, et al. 2020; Hacker et al. 2023; Hacker and Graham 2023), they are less likely to approach food when a person is looking at them (Goumas et al. 2019) and they kleptoparasitise more in urban areas than rural areas (Spencer et al. 2017). Sensitivity to gaze direction is present in juvenile and adult gulls in both rural and urban areas, and thus does not appear to require extensive experience with humans (Goumas, Collins, et al. 2020; Lamond and Fisher 2023). We tested whether eye‐like stimuli could be used as an effective, non‐invasive deterrent on human food packaging in the UK, to mitigate conflict due to scavenging and stealing food in this species of conservation concern.

We presented herring gulls in towns with pairs of takeaway food boxes. These boxes, commonly used throughout the UK for ‘fast’ food that is often eaten outside (such as burgers, fish and chips), represent objects that gulls may investigate as potential food sources because gulls have the opportunity to observe humans eating from them (Goumas, Boogert, et al. 2020; Hacker et al. 2023; Hacker and Graham 2023). One box was plain and the other box had a pair of eye‐like stimuli stuck on each surface, and we recorded latency to approach and peck at either box. We then tested for short‐term habituation to repeated exposure to stimuli by presenting either the plain box or the box with eyes to individual gulls over three trials. We expected that gulls would be more likely to approach and peck at the box without eyes, but that gulls would become less wary of boxes with eyes during subsequent presentations. We then tested whether resemblance to eyes or stimulus contrast could explain aversive responses by presenting gulls with pairs of takeaway boxes that had either high‐contrast eye‐like circles or high‐contrast squares on the surface. If eye resemblance is an important deterrent mechanism in this species, we expected that boxes with circular stimuli would be approached more slowly and pecked less than boxes with square stimuli.

## 2 Methods

### 2.1 Test Subjects

**FIGURE 1:**
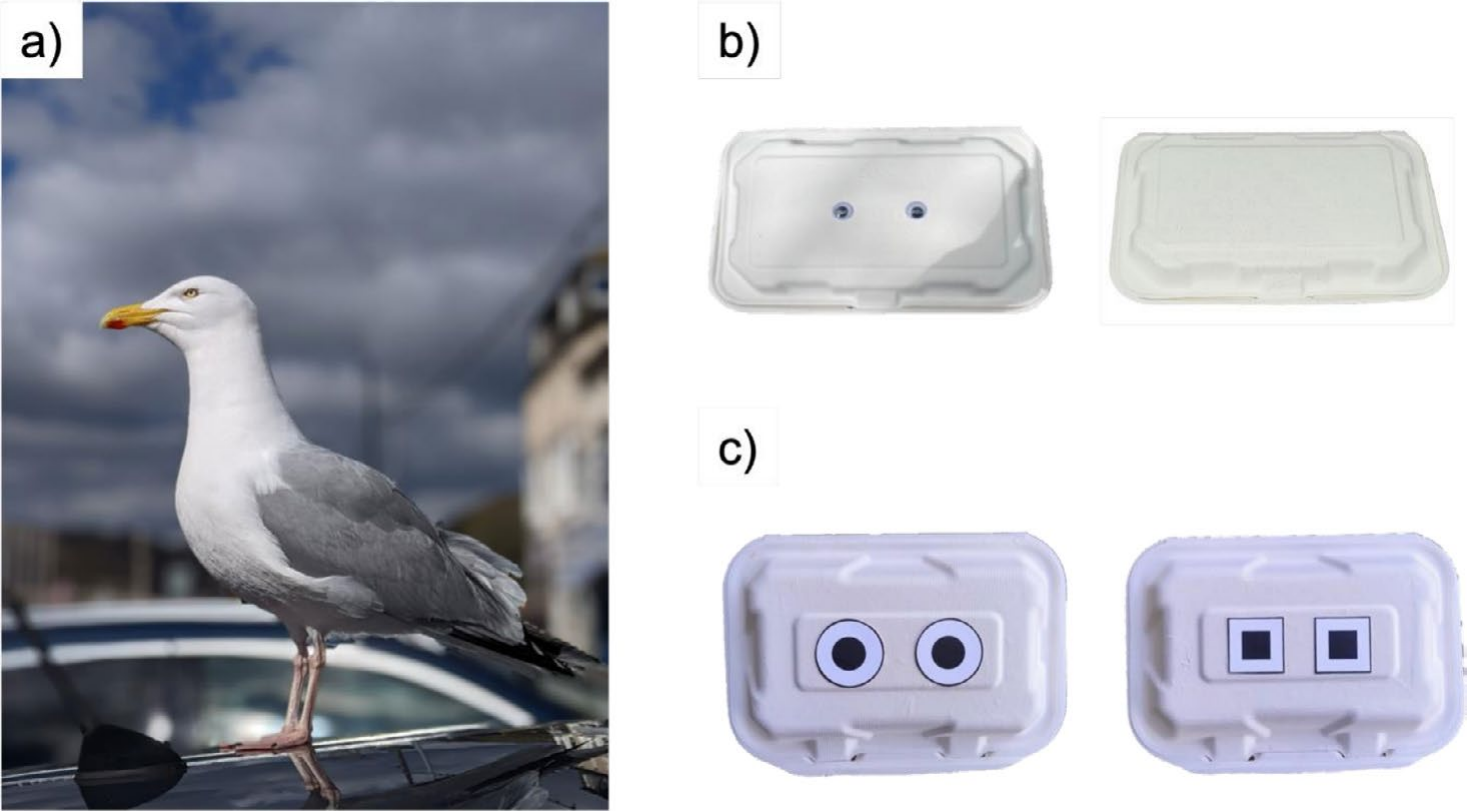
(a) adult European herring gull (photo courtesy of Céline Rémy); (b) boxes presented in eyes versus plain choice experiment. Box on left has ‘googly’ eyes on each surface although only the eyes on the uppermost surface were visible to approaching gulls; (c) boxes presented in the high contrast experiment, where box on the left has circular high contrast stimuli and box on the right has square high contrast stimuli.

We tested herring gulls (Figure 1a) in coastal towns in Cornwall and Devon, UK. During spring and summer, individual gulls or mated pairs inhabit territories from which they chase away intruders (Drury and Smith 1968). Although we could not reliably identify individual birds, we tried to minimise the risk of testing the same individual multiple times by running trials at least 20 m apart when testing in the same town, and used features such as size, age class and plumage details to try and discriminate the focal bird from other gulls in the area. We tested both adult and immature gulls, the latter being distinguishable by the presence of brown plumage. Two experimenters were present during all data collection.

### 2.2 Eyes Versus Plain Choice Experiment

Experiments were conducted in May and June 2022 in six towns in Cornwall. Once a gull was located, an experimenter sat down approximately 5 m away with two closed white clamshell bagasse takeaway food boxes (229 × 152 × 76 mm; Ecoware UK) on their lap. One box was plain and the other had two eyes attached to each outer surface (Figure 1b). We used ‘googly’ eyes (Toaob, China) as stimuli, where each eye was a clear plastic dome with a loose flat black ‘pupil’ disk inside (eyes were 15 mm in diameter and placed 35 mm apart, hereafter referred to as ‘eyes’). When the gull demonstrated interest (orienting head or body towards the experimenter or flying to the ground), the experimenter started gently throwing 20 mm^2^ pieces of sliced white bread towards the gull. After three pieces of bread were thrown, the experimenter simultaneously placed the boxes on the ground 2 m apart (as measured by outstretched arms on either side). Prior to the experiment, each box was randomly assigned to the left or right side for each trial using the sample function in base R (R‐Core‐Team 2025). Both boxes were filled with rocks to weigh them down but contained no food. After placing the boxes on the ground, the experimenter stood up and walked away slowly to a video camera located at least 2 m directly behind the boxes where the observer was recording and started a timer when the gull was within a 3 m radius of the box. Distances were estimated by eye, by the same person throughout for consistency. Gull behaviour was observed for 5 min; if the gull pecked at one of the boxes the experimenter approached the boxes and picked them up to end the trial. If the gull did not peck at either box within 5 min, the trial was deemed unsuccessful and excluded from analysis. Both experimenter and observer wore sunglasses for the duration of the experiment to reduce the availability of human visual cues. The observer recorded which box was pecked first and latency to peck. Choice was defined as the box pecked first by a gull and latency was the duration from the start of the trial (i.e., boxes being placed on the ground) to the first peck at a box.

### 2.3 Habituation Experiment

Experiments were conducted in June and July 2022 in four towns across Cornwall. The protocol and stimuli were the same as in the previous choice experiment, but only one box (plain or box with eyes) was placed 1 m in front of the experimenter. Prior to the experiment, the box presented in each trial was selected randomly using the sample function in base R, so that each box treatment (plain or with eyes) was presented to exactly 15 gulls (i.e., hypergeometric sampling). A timer was started once the box had been placed on the ground and the experimenter had retreated to the observer and video camera at least 2 m away. After the gull had pecked the box or 5 min had passed, the experimenter retrieved the box. After a 1‐min interval, the experimenter repeated the trial on the same gull using the same box and throwing the gull three pieces of bread at the start of the trial. This was then repeated for a third time, so that each focal gull experienced three presentations of the same box. If the gull did not peck at the box within 5 min (and showed interest in the box throughout the trial), then a time of 5 min was recorded for latency to approach, so the durations recorded represented the minimum number of seconds until a box was pecked. We did not eliminate these data points from analysis as some gulls who failed to peck in earlier trials did so in later trials and vice versa.

### 2.4 High Contrast Choice Experiment

Gulls were tested in coastal towns in Cornwall and Dorset between August and November 2021. The protocol was similar to the eyes versus plain box experiment above, where a gull was simultaneously presented with two closed clamshell bagasse takeaway boxes (220 × 152 × 70 mm; PBK Products UK, Figure 1c) placed 2 m apart on the ground by an experimenter, and the latency to approach and peck was recorded. In this set of trials, both boxes had one pair of stimuli 20 mm apart (edge to edge) on the top surface of the box—either white circles with black centres (eye‐like stimuli) or white squares with a black central square (non‐eye‐like contrast matched). Circles and squares were matched for total area of black and white (white area = 914 mm^2^, black area = 311 mm^2^, diameter of circle stimulus = 40 mm, diameter of square stimulus = 35 mm). Trials ran for 10 min and a trial was deemed unsuccessful and excluded from the analysis if the focal gull did not approach or peck at either box within this time. We used a longer trial time compared with the previous two experiments to determine whether gulls would be more likely to peck at a stimulus if given a longer exposure time.

### 2.5 Statistical Analysis

All data were analysed in R using dplyr, ggplot2, lme4, survival, coxme and survminer packages for data manipulation, visualisation and statistical testing (Wickham et al. 2023; Wickham 2016; Bates et al. 2015; Therneau 2024a, 2024b; Kassambara et al. 2021).

### 2.6 Eyes Versus Plain and High Contrast Experiments

To test whether gulls showed aversion to eye‐like stimuli on boxes, we carried out binomial tests for each set of binary choice experiments to assess whether gulls were less likely to peck at a box with eye‐like stimuli compared to a control box (either plain or square stimuli). We then tested whether birds were also slower to approach boxes with eye‐like stimuli compared with control boxes. We used two linear models, one for the eyes versus plain experiment where the control was a plain box and one for the contrast experiment where the control was square stimuli. In both models, time to peck chosen box was the response variable and first box pecked was the predictor variable and assumptions of normality were met.

### 2.7 Habituation Experiment

There were multiple trials where the focal gull did not peck during the 10 min of the experiment, so we used a survival analysis, censoring values where gulls did not peck within the trial. We used a Cox mixed effects model as assumptions surrounding proportional hazards were met. The duration until a box was pecked was the response variable, and box type (eyes or plain), trial number (1, 2 and 3) and their interaction were fixed effects. Individual gull was included as a random effect.

### 2.8 Ethics

This work was approved by the University of Exeter Ethics Committee (4874063) and adhered to the ASAB/ABS Guidelines for the Use of Animals in Research.

## 3 Results

### 3.1 Eyes Versus Plain Box Choice Experiment

**FIGURE 2:**
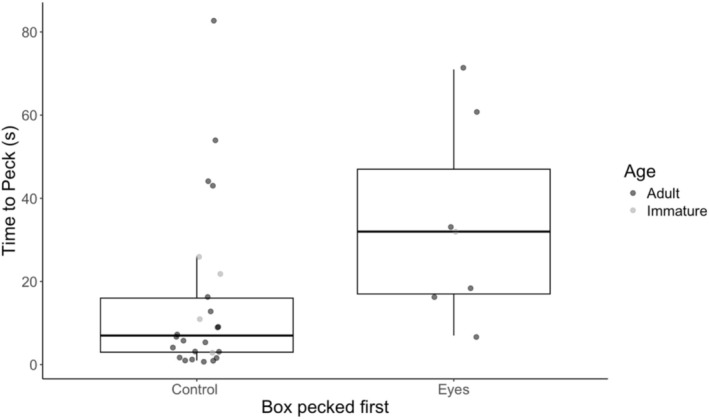
Latency to peck boxes without eyes (control) and with eyes. Boxplots show medians, upper and lower quartiles, with whiskers showing maximum and minimum values. Raw data points for adults are shown in dark grey and for immatures in light grey.

We tested 41 herring gulls across all locations and of these, 32 (78%) pecked a box within 5 min, comprising 27 adults and five immatures. The box with eyes on was pecked significantly less (7/32 trials; 22%) than the plain control box (25/32; 78%; exact binomial test = 0.22; 95% CI 0.09–0.40, *p* = 0.002), which did not change when only adults were considered (6/27 trials, exact binomial test = 0.22, 95% CI 0.09–0.42; *p* = 0.006). The sample size was too small to test immatures, but only one immature bird of five tested pecked at the box with eyes. Gulls also took significantly longer to peck the eye box than the control box (linear model; *t* = 2.10, *F*_1,30_ = 4.39, *p* = 0.04, *R* ^2^ = 0.10; Figure 2). The eye box was pecked on average 19 s slower than the control box (eyes 34.0 ± 23.8 s, control 15.0 ± 20.4 s, mean ± SD).

### 3.2 Short‐Term Habituation Experiment

**FIGURE 3:**
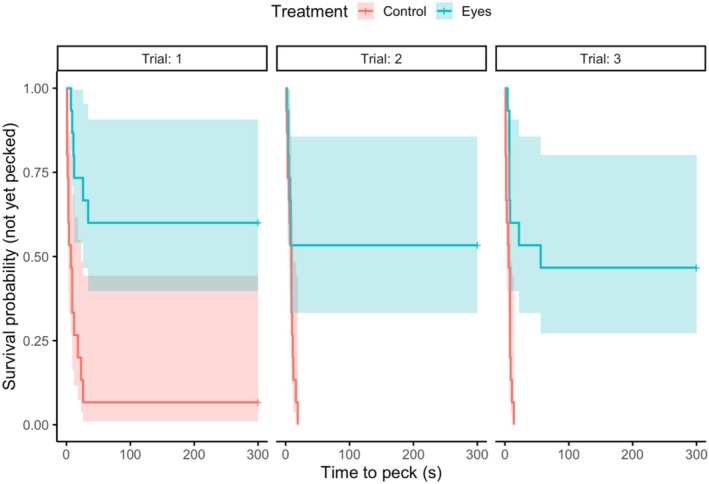
Kaplan–Meier curve showing likelihood of gull pecking at plain control box (red line, *n* = 15) and box with eyes (blue line, *n* = 15) over 10 min trials, with three repeated presentations (trial 1, 2 and 3). Shaded areas show 95% confidence intervals.

Of 38 gulls that were presented with a box, 30 gulls (79%) were tested three times, 15 gulls on plain control boxes and 15 on boxes with eyes. The control box was pecked significantly more (44/45 presentations, 98%) than the eye box (21/45 presentations, 47%; Fisher's exact test *p* < 0.001). Seven of the 15 birds tested (47%) never pecked at the eye box over all three trials. A mixed‐effects Cox proportional hazards model revealed a significant interaction between stimulus type and trial (treatment × trial 2: *β* = 2.07, SE = 0.79, *z* = 2.61, *p* = 0.009; treatment × trial 3: *β* = 1.19, SE = 0.74, *z* = 1.60, *p* = 0.109; Table 1), indicating that the treatment effect changed across repeated exposures (see Figure A1 in appendix). In trial 1, birds were substantially slower to peck the eyes box compared to the plain control box (HR = 0.02, Figure 3). This avoidance was reduced by trial 2 (HR = 0.17), indicating an increase in pecking hazard. By trial 3, the hazard ratio remained below 1 (HR = 0.07), but the trial 3 interaction was not significant. Gulls were less likely to peck at the box with eyes (Table 1).

**TABLE 1:** Output of Cox proportional hazards model investigating effect of box type (eyes or no eyes) over three presentations (trials) on maximum time to peck at box.

Predictor	*β* (coef)	Hazard ratio	SE	*z*	*p*
Treatment	−3.84	0.02	0.96	−4.01	< 0.001
Trial 2	−0.15	0.86	0.43	−0.35	0.73
Trial 3	0.58	1.79	0.41	1.41	0.16
Treatment*Trial 2	2.07	7.91	0.79	2.61	0.009
Treatment*Trial 3	1.19	3.29	0.74	1.60	0.11

*Note:* Random effect bird ID (SD = 1.92, variance = 3.70).

### 3.3 High Contrast Experiment

**FIGURE 4:**
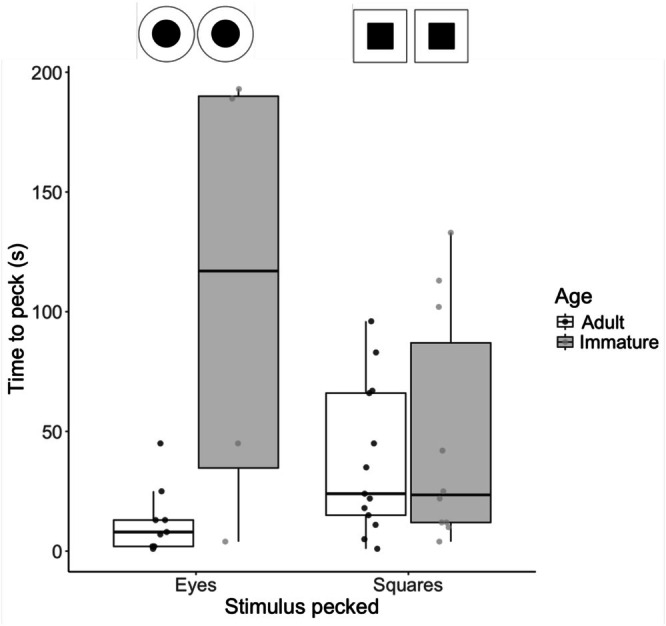
Boxplots showing the latency for adults (white) and immature birds (grey) to peck boxes with eyes or squares. Stimuli are shown at the top (not to scale). Boxplots show medians, upper and lower quartiles, with whiskers showing maximum and minimum values. Raw data points are shown in dark grey (adults) and light grey (immatures).

We presented 46 herring gulls with two boxes simultaneously; one box that had pairs of circular (eye‐like) stimuli and the other box with square (control) stimuli. A total of 36 gulls (78%) pecked at a box. Of these birds, 22 were adults and 14 were immatures, so we analysed results for these two age groups both separately and pooled together. The box with eye‐like stimuli was pecked less (13/36 trials; 36%) than the square stimuli control box (23/36; 64%). However, this difference was not significant, irrespective of whether gulls were analysed separately by age or pooled together (exact binomial test of pooled data = 0.36, 95% CI 0.21–0.54, *p* = 0.13). Although the small sample size could not accommodate a test for an interaction between age and treatment, immature birds appeared to take longer to peck a box with eyes compared to a box with squares (Figure 4). There was no difference in latency to peck across stimulus type (stimulus type *t*_2,33_ = −0.30, *p* = 0.77), but adult gulls were faster to peck (age *t*_2,33_ = 2.28, *p* = 0.03; Figure 4).

## 4 Discussion

We tested whether eye‐like stimuli were aversive to herring gulls foraging in towns. We found that some gulls were slower to approach and peck at a takeaway box with ‘googly’ eyes than a box without eyes. The aversive effect of eyes seemed to vary across individuals, where approximately half of the birds tested over multiple trials never approached a box with eyes, whereas the other half approached and pecked at boxes with eyes at a similar speed to gulls presented with boxes without eyes. These results suggest strong initial avoidance of the eyes stimulus for some gulls, followed by partial habituation over subsequent trials for those who pecked/engaged at all. We also found that there was no difference in gull latency to approach and peck at boxes with high contrast stimuli that differed in their shape. Therefore, it seems likely that high contrast black and white stimuli were primarily responsible for eliciting aversive behaviour, rather than resemblance to eyes per se.

Our results suggest that high contrast stimuli such as eyespots can deter some gulls upon initial encounter. This deterrent effect is consistent with studies across a wide range of animals in foraging and nest‐site searching contexts (Radford et al. 2020; Fukuda et al. 2008; Rouxel et al. 2021; Davidson et al. 2014; Halpin et al. 2020; Skelhorn and Rowland 2022; De Bona et al. 2015; Montevecchi et al. 2023). For example, eye‐like markings decreased crop loss from foraging starlings *Sturnus vulgaris* (Fukuda et al. 2008). However, the efficacy of high contrast or eye‐like stimuli may vary due to environmental factors and the species targeted. For example, bycatch of diving seabirds from gillnet fisheries can be reduced by the presence of eye‐like stimuli and high‐contrast banners in some locations (Rouxel et al. 2021; Montevecchi et al. 2023), but not others (Rouxel et al. 2023). In this study, gulls simultaneously presented with boxes both with and without eyes were more likely to peck at the box without eyes and were faster to peck at these boxes. Although a 19 s difference in pecking time is relatively small, it provides enough time for a person to notice an approaching gull and take appropriate countermeasures, potentially preventing kleptoparasitic events.

Gulls appeared to respond to repeated short term presentation of eye‐like markings in one of two ways—they either approached and pecked at a similar speed to boxes without eyes, or they did not approach at all, even after repeated presentations. When gulls did peck at boxes with eyes, the time to peck either remained the same or decreased with subsequent presentations i.e., the time to peck never increased. We did not have a large enough sample size to test the effect of location on the deterrent effect of eye‐like markings. Gulls show sensitivity to human eye gaze direction in both urban and rural areas (Goumas, Collins, et al. 2020; Goumas et al. 2019), but future studies could consider whether the effectiveness of eye‐like stimuli may vary with context as seen in other studies (Rouxel et al. 2021, 2023). Contrary to popular belief, many herring gulls are wary of approaching novel objects or humans (Goumas, Boogert, et al. 2020; Goumas et al. 2019; Goumas, Collins, et al. 2020; Inzani et al. 2023). We may have tested gulls that were generally bold with low neophobia in urban areas, as these were more likely to participate in experiments. However, as these individuals are likely to engage in behaviours that bring them into conflict with humans, such as food snatching, they would also be the targets of deterrent measures.

It would be useful to determine whether wary individuals can overcome aversion to eye‐like stimuli after repeated exposure. We found some evidence to suggest that immature gulls may find eye‐like stimuli aversive, as they were generally slower to approach circular high‐contrast stimuli compared with square stimuli. However, our sample size was too small to be conclusive and future work could investigate whether the effectiveness of eye‐like and high contrast stimuli declines with experience. Previous studies have shown that habituation to eye‐like stimuli can be low after days or weeks of repeated exposure (Fukuda et al. 2008; Rouxel et al. 2021), but it is unknown whether individual birds were repeatedly deterred or whether different birds were deterred by the stimuli during the course of those trials.

In this study 64% of gulls pecked at a box with square stimuli compared with 36% with round stimuli, which may indicate that circular stimuli are more aversive and that we lacked the statistical power needed to detect this effect. However, both eye‐like and high contrast stimuli may be effective deterrents because they exploit pre‐existing sensory biases to evoke an aversive response (Stevens 2005). As a result, stimuli may not need to resemble eyes closely—previous studies have used eye‐like stimuli similar to those shown in figure 3, presented on balloons (Fukuda et al. 2008), rotating panels (Rouxel et al. 2021), paper (Davidson et al. 2014; Stevens et al. 2008; Skelhorn and Rowland 2022) and computer monitors (De Bona et al. 2015), which are unlikely to be perceived as belonging to a predator. The gulls in our current study are unlikely to have perceived the eyes on boxes as the eyes of another animal, because they were clearly not part of a predator's body. The eye‐like stimuli and high contrast stimuli used here, and in similar studies, may be particularly salient because they comprise a dark central region with a light surrounding area, which maximally stimulate vertebrate receptive fields in the retina and associated brain regions (Stevens 2005; Stevens et al. 2008). The square stimuli we used may also appear relatively eye‐like given they have the same general pattern of a black centre surrounded by white. It would be interesting to test gull responses to other high contrast stimuli that lack this visual pattern, for example black and white stripes or dots. Despite evidence of the efficacy of eye‐like stimuli in deterring foraging animals, the underlying mechanisms are still poorly understood. For example, eyespot size has been shown to affect avian predation rate in some butterflies (Stevens et al. 2008; Ho et al. 2016; Kodandaramaiah et al. 2013) and it remains to be explored whether larger high‐contrast stimuli that do not resemble eyes may be more aversive than smaller stimuli. The importance of arranging stimuli in pairs also deserves further attention, as paired stimuli, whether eye‐like or not, can be more aversive to an avian predator than non‐paired stimuli, irrespective of the number of pairs present (Mukherjee and Kodandaramaiah 2015).

Identifying and exploiting pre‐existing sensory biases of animals remains an important area of research for animal deterrents. Previous studies have used visual deterrents such as eye‐like stimuli, high contrast patterns and predator silhouettes to reduce seabird bycatch from gillnets, with some success (Rouxel et al. 2021; Montevecchi et al. 2023; Almeida et al. 2023). Whilst most research into eye‐like and high‐contrast stimuli has been focussed on avian deterrents, they can also be effective in other animals due to general sensitivity to eye‐like stimuli. Eye‐like and high‐contrast stimuli painted on cattle rumps reduced attacks by ambush carnivores (Radford et al. 2020), and eye‐like stimuli can slow down or divert attacks in fish (Kjernsmo and Merilaita 2017, 2013). In contexts where the efficacy of these stimuli may be variable due to differences among individuals or species, or reduced due to prolonged exposure, rotating the use of multiple deterrents (either a different visual stimulus or a deterrent in a different sensory modality) may help to increase the efficacy of deterrents, reduce habituation and offer a low‐cost, non‐lethal way of deterring animals (Laguna et al. 2022; Rutherford et al. 2025; Biedenweg et al. 2011). An effective gull deterrent could incorporate short‐term exposure to large eye‐like stimuli accompanied by an acoustic deterrent such as gull alarm calls or human voices (Di Giovanni et al. 2022; Rémy et al. 2025), to maximise the deterrent effect whilst minimising the likelihood of habituation. However, it is crucial to consider behavioural differences among individuals when assessing the effectiveness of deterrents. Sensory deterrents are also one part of a broader set of measures needed to reduce human‐gull conflict, including public information discouraging feeding gulls and improved food waste management (Calladine et al. 2006).

## Author Contributions

**Laura A. Kelley:** conceptualization (equal), formal analysis (equal), funding acquisition (equal), investigation (equal), project administration (equal), supervision (equal), writing – original draft (lead). **Rosa Hunter Thompson:** data curation (equal), formal analysis (supporting), investigation (equal), methodology (equal), visualization (supporting), writing – review and editing (supporting). **Beth Rowe:** data curation (equal), formal analysis (supporting), investigation (equal), visualization (supporting), writing – review and editing (supporting). **Neeltje J. Boogert:** conceptualization (equal), funding acquisition (equal), methodology (equal), project administration (equal), supervision (equal), writing – review and editing (equal).

## Funding

L.A.K. and N.J.B. are funded by Royal Society Dorothy Hodgkin Research Fellowships (L.A.K. Grant DH160082 and N.J.B. Grant DH140080).

## Conflicts of Interest

The authors declare no conflicts of interest.

## Supporting information


**Figure A1.** Boxplots showing maximum time to peck at a plain control box (*n* = 15) and a box with eyes (*n* = 15). Boxplots show medians, upper and lower quartiles, with whiskers showing maximum and minimum values. Dotted lines join points of individual birds (indicated by different colours) across trials.

## Data Availability

Data and R code are available at https://figshare.com/s/02eb8d28a921d5d4d2ec.
