# Supplementary material for: Evaluating Aversion to Eye‐Like Stimuli as a Foraging Deterrent in Urban European Herring Gulls
Source: Ecol Evol. 2026 Mar 8;16(3):e73202. doi: 10.1002/ece3.73202 (PMC12967596; doi:10.1002/ece3.73202)
Supplement: Supplementary file 1 — Figure A1. Boxplots showing maximum time to peck at a plain control box (n = 15) and a box with eyes (n = 15). Boxplots show medians, upper and lower quartiles, with whiskers showing maximum and minimum values. Dotted lines join points of individual birds (indicated by different colours) across trials. [file ECE3-16-e73202-s001.docx]

Appendix


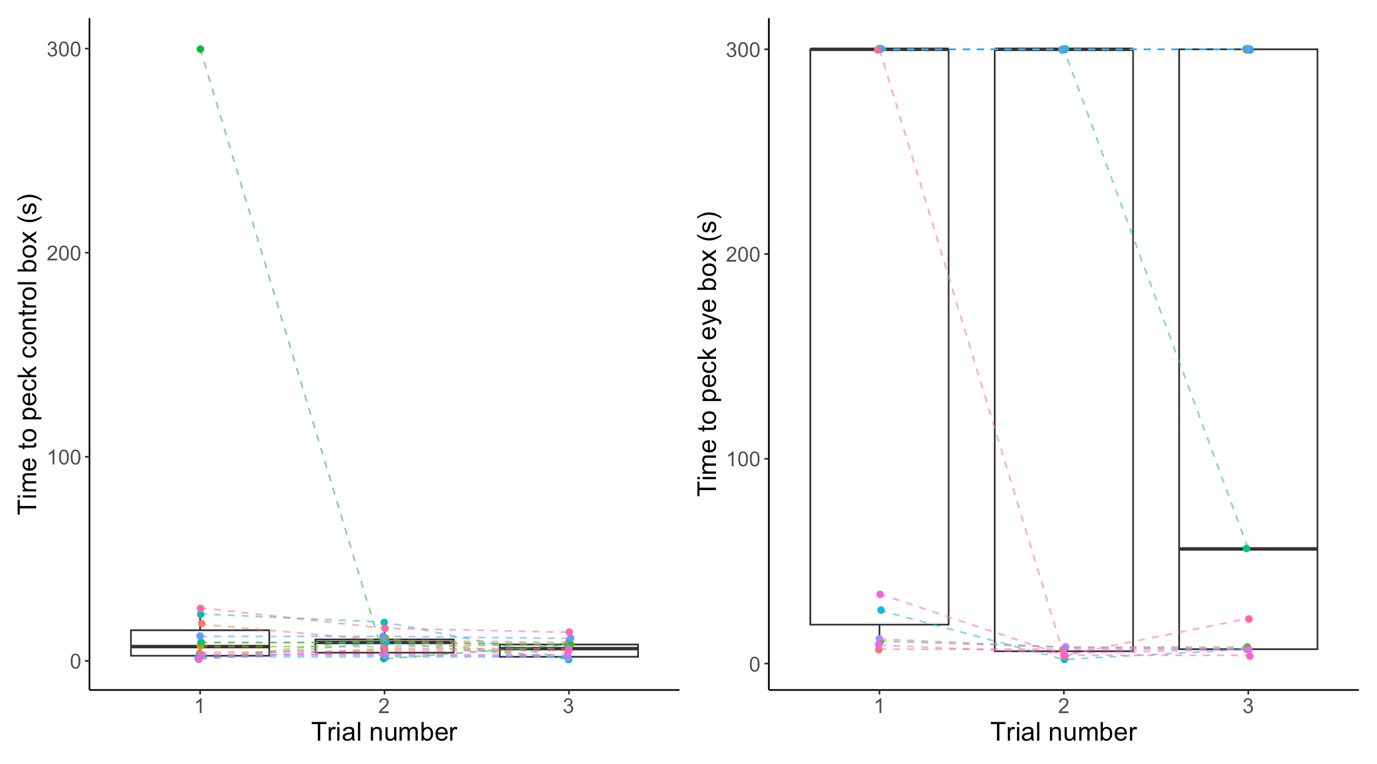


Figure A1. Boxplots showing maximum time to peck at a plain control box (n = 15) and a box with eyes (n = 15). Boxplots show medians, upper and lower quartiles, with whiskers showing maximum and minimum values. Dotted lines join points of individual birds (indicated by different colours) across trials.
